# ACE2 Protein Landscape in the Head and Neck Region: The Conundrum of SARS-CoV-2 Infection

**DOI:** 10.3390/biology9080235

**Published:** 2020-08-18

**Authors:** Géraldine Descamps, Laurine Verset, Anne Trelcat, Claire Hopkins, Jérome R. Lechien, Fabrice Journe, Sven Saussez

**Affiliations:** 1 Department of Human Anatomy and Experimental Oncology, Faculty of Medicine and Pharmacy, UMONS Research Institute for Health Sciences and Technology, University of Mons (UMONS), Avenue du Champ de Mars 6, 7000 Mons, Belgium; geraldine.descamps@umons.ac.be (G.D.); anne.trelcat@umons.ac.be (A.T.); jerome.lechien@umons.ac.be (J.R.L.); fabrice.journe@umons.ac.be (F.J.); 2 Department of Pathology, Institute Jules Bordet, Université Libre de Bruxelles, Rue Héger-Bordet 1, 1000 Brussels, Belgium; laurine.verset@bordet.be; 3 Guy’s and St Thomas’ Hospitals, Westminster Bridge Road, London SE1 9RT, UK; clairehopkins@yahoo.com; 4 British Rhinological Society (President), 35-43 Lincoln's Inn Fields, London WC2A 3PE, UK; 5 Department of Otolaryngology-Head & Neck Surgery, Foch Hospital, School of Medicine, UFR Simone Veil, Université Versailles Saint-Quentin-en-Yvelines (Paris Saclay University), 40 Rue Worth, Suresnes, 92150 Paris, France; 6 Department of Otorhinolaryngology and Head and Neck Surgery, CHU de Bruxelles, CHU Saint-Pierre, Université Libre de Bruxelles, Rue aux Laines 105, 1000 Brussels, Belgium; 7 Department of Oncology and Experimental Surgery, Institute Jules Bordet (IJB), Université Libre de Bruxelles (ULB), Rue Heger-Bordet 1, 1000 Brussels, Belgium

**Keywords:** ACE2, head and neck, SARS-CoV-2, immunohistochemistry, protein

## Abstract

The coronavirus pandemic raging worldwide since December 2019 is caused by the severe acute respiratory syndrome coronavirus 2 (SARS-CoV-2), which invades human cells via the angiotensin-converting enzyme 2 (ACE2) receptor. Although it has already been identified in many organs, ACE2 expression remains largely unknown in the head and neck (HN) sphere. Thus, this study aims to investigate its protein expression in several sites of the upper aerodigestive tract in order to highlight potential routes of infection. We compared ACE2 immunohistochemical expression between 70 paraffin-embedded specimens with two different antibodies and reported the quantified expression in each histological location. Surprisingly, we obtained different results depending on the antibody, an absence of labeling having been observed with a monoclonal antibody raised against the extracellular domain, whereas the polyclonal, against the cytoplasmic part of the protein, revealed enriched ACE2 expression, particularly in sinuses, vocal cords, salivary glands and oral cavity epithelial cells. The interpretation of these discordant results has brought several exciting lines of reflection. In conclusion, this study provides possible routes of entry for the SARS-CoV-2 in HN region and, above all, has led us to encourage caution when studying the ACE2 expression which is currently at the center of all attention.

## 1. Introduction

In December 2019, the cradle of an unprecedented new respiratory syndrome outbreak took its origin in Wuhan, China and was quickly attributed to a new β-coronavirus identified as SARS-CoV-2 by the International Committee on Taxonomy of Viruses, giving rise to what is more commonly known as coronavirus disease 2019 (COVID-19) [1,2]. To date, this highly contagious disease has infected 8,708,008 citizens, with 461,715 corresponding deaths [3] (WHO Situation Report 06/21/2020) and spread to 203 countries in 5 months, which consequently earned it the inglorious title of a pandemic, as declared by the Director-General of the World Health Organization on March 11, 2020. The symptomatic pattern of COVID-19 is relatively broad and can range from asymptomatic upper respiratory tract infection to critical acute respiratory distress syndrome which can finally lead to death [3,4,5].

Following the epidemic of SARS-CoV in 2003, ACE2 was identified as being the functional receptor necessary for the entry of this pathogen into host cells [6]. The similarities between SARS-CoV and SARS-CoV-2 prompted researchers to investigate the role of ACE2 in the initiation of this multi-symptomatic disease, suspecting a strong binding affinity between this new virus and ACE2 as a receptor. Indeed, genomic analyses have recently demonstrated a homology of almost 80% between the two viruses, suggesting that they may infect human cells through the binding of the same ACE2 receptor [7,8]. It would not take long to assert the importance of ACE2 as a gateway for this new pathogen. Since the results recently published by many teams, there is no longer any doubt that SARS-CoV-2 depends on the ACE2 receptor to penetrate human cells [9,10,11,12], making this receptor a major focus of this public health debate.

ACE2 consists of 805 amino acids and weighs approximately 120 kDa. It is a type I transmembrane glycoprotein which has an aminoterminal catalytic extracellular domain of 740 aminoacids and a carboxyterminal one comprising 43 aminoacids. In addition to its carboxypeptidase functions on Angiotensin I and II, ACE2 exerts beneficial regulatory effects in many organs such as the heart, kidneys and lungs [13], which are also the organs where the symptoms arise after infection with the virus. Indeed, the wide range of ACE2 expression also explains the extra-pulmonary symptoms seen in COVID-19. It is particularly expressed in vascular endothelial cells, renal tubular cells, alveolar epithelial cells of the lung, enterocytes of the intestine, myocardial cells and in the smooth muscle cells, which underlines the potential routes of infection and spread of the virus [14,15].

Although documented in many organs, the expression of ACE2 remains poorly investigated in the upper aerodigestive tract while these anatomical regions are at the forefront when the virus enters, given the spread by respiratory droplets [3]. In fact, many otorhinolaryngological symptoms have been described in patients with moderate COVID19 infection, particularly a sudden loss of smell and gustatory dysfunctions [16,17]. Interestingly, Xu et al. recently demonstrated the expression of ACE2 in oral mucosa, specifically in epithelial cells, and to a lesser extent in T cells, B cells, and fibroblasts through single-cell RNA-seq of carcinomas and normal tissues listed from public databases, highlighting a new potential route of SARS-CoV-2 infection [18]. Finally, ACE2 expression has also been reported in the salivary gland ducts of Rhesus macaques, supporting (i) a possible additional organ target for COVID19, (ii) growing interest on saliva for the diagnosis of the disease, and (iii) the concept that asymptomatic people could spread the infection through saliva [19,20].

To better understand the pathogenesis and the potential routes of infection in head and neck regions, we explored the immunolocalization of ACE2 protein in seven different tissue types and identified cell types expressing ACE2.

## 2. Material and Methods

### 2.1. Human Tissue Specimens

All specimens used in this study were obtained from patients who received information regarding the use of residual human corporal materials for clinical research. This study was conducted according to the national ethical guidelines, and the Institutional Review Board approved the procedure (IJB-CE3157).

Human tissue biopsies from seven different location in the upper aerodigestive tract were obtained from patients who underwent surgery in several Belgian hospitals for various reasons, such as sleep surgery, chronic rhinosinusitis, or for cancer resection. Among samples from patients with cancer, only normal peritumoral epithelia were analyzed. The anatomical origin of tissues was chosen to investigate the potential entry route of SARS-CoV-2 in humans. Tissues were analyzed from 70 different specimens, as described in Table 1. Each anatomical location is represented in the Figure S1. The kidney and small intestine tissues were used as positive and negative controls.

### 2.2. Immunohistochemistry and ACE2 Scoring

**Figure 1:**
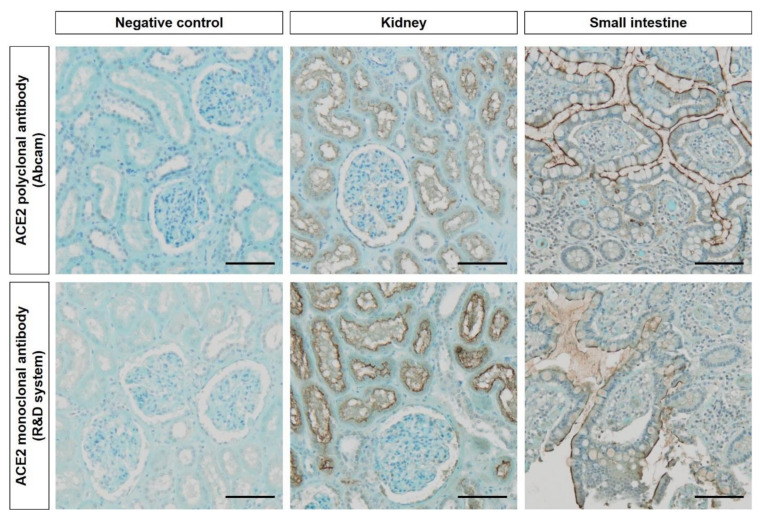
Negative and positive controls for ACE2 used for both antibodies. Kidney tissues were used as negative controls and kidney or small intestine tissues for positive controls. Scale bar = 100 µm.

All tissue samples were fixed in 4% buffered formaldehyde for 24 h, dehydrated and embedded in paraffin. Immunohistochemistry was performed on 5-µm-thick sections mounted on silane-coated glass slides. After deparaffinization with xylene and rehydration with decreasing concentrations of ethanol, sections were incubated with 4.5% hydrogen peroxide in distilled water for 10 min to block endogenous peroxidase activity and rinsed in distilled water. Tissues were then heated in a pressure cooker for 6 min to induced antigen retrieval in a 0.01 M citrate buffer solution (pH 6.2) (Scytek, West Logan, UT, USA). After thorough washing with phosphate-buffered saline (PBS), the sections were incubated for 30 min in a solution of 0.5% casein in PBS and exposed to ACE2 primary antibodies. The polyclonal rabbit anti-ACE2 (ab15348, Abcam, Cambridge, UK) and the monoclonal mouse anti-ACE2 (MAB933, R&D systems, Minneapolis, MN, USA) were diluted (1:1500 and 1:50, respectively) in PBS with 0.05% casein, were applied on sections and incubated overnight at 4 °C. Subsequently, the samples were incubated with a PowerVision Poly HRP-anti-rabbit and anti-mouse IgG (Klinipath, Duiven, Holland), and the antigens were visualized via the addition of a solution of 3-3′-diaminobenzidine tetrachloride (DAB)—H_2_O_2_ buffer (Liquid DAB, San Ramon, CA, USA) before being counterstained with Mayer’s hemalum (Klinipath, Duiven, Holland) and Luxol fast blue and finally mounted with a synthetic balm (Thermo Scientific, Pittsburg, PA, USA). To exclude antigen-independent staining, negative controls for which the incubation step with the primary antibody was omitted were examined. In all instance, these controls did not reveal any staining. ACE2 immunoreactivity was initially detected in positive controls (kidney and small intestine tissues) known to express ACE2 (The Human Protein Atlas) (Figure 1).

The slides were analyzed using a BX43 Olympus microscope. The percentage of stained cells and staining intensity were semi-quantitatively assessed by one pathologist (LV) using a 4-grade scale for both parameters. The percentage of positivity was described as 0 = no stained cells, 1 = less than 25%, 2 = between 25 to 75%, 3 = above 75%. The intensity of the staining is counted as 0 = no expression/intensity, 1 = weak intensity, 2 = moderate, 3 = strong). Positivity in inflammatory cells and in fibroblasts were assessed as present or absent. An immunostaining score was finally calculated by the sum of these two values (percentage + intensity) and a value from 0 to 6 was attributed to each cell type, and tissue location analyzed.

### 2.3. Statistical Analyzes

The median scores of the independent data groups were compared by using nonparametric Kruskal–Wallis test for the epithelial cell components. Association between ACE2 score of epithelial cells and clinical data of patients was evaluated by Mann–Whitney test. Correlation between immunostaining score of epithelial cells and age of patients was assessed by Spearman’s Rho test. All statistical analyses were performed using the SPSS Statistics 23 software (IBM, Ehningen, Germany) and a *p*-value <0.05 was considered to be significant.

## 3. Results

Recently, numerous transcriptomic and RNAseq studies have evaluated ACE2 expression at the mRNA level. To confirm and provide further information at the protein level, we performed immunohistochemistry with two antibodies, a polyclonal and a monoclonal, raised against, respectively, the intracellular and extracellular part of the protein, to visualize ACE2 protein localization in different anatomical sites of the upper aerodigestive tract. This approach also provides additional spatial information, which is important for understanding the possible ACE2 cellular interaction sites for COVID-19.

### 3.1. ACE2 Expression in Control Tissues

As recommended, we developed antibody adjustments on human small intestine and kidney samples as positive controls. Figure 1 shows distinct ACE2 expression pattern in both organs using the two independent antibodies as well as negative controls. In the kidney, moderate to strong ACE2 staining was detected in the tubular cells without glomerular expression. The expression in the small intestine was precisely and abundantly localized in the brush border of enterocytes for both antibodies (Figure 1). These results confirmed the observations reported by The Human Protein Atlas and by relevant publications [21,22], so we performed the same immunohistochemical analyzes throughout the upper aerodigestive tract, from nasal cavity to hypopharynx.

### 3.2. ACE2 Expression in Epithelial Cells

**Figure 2:**
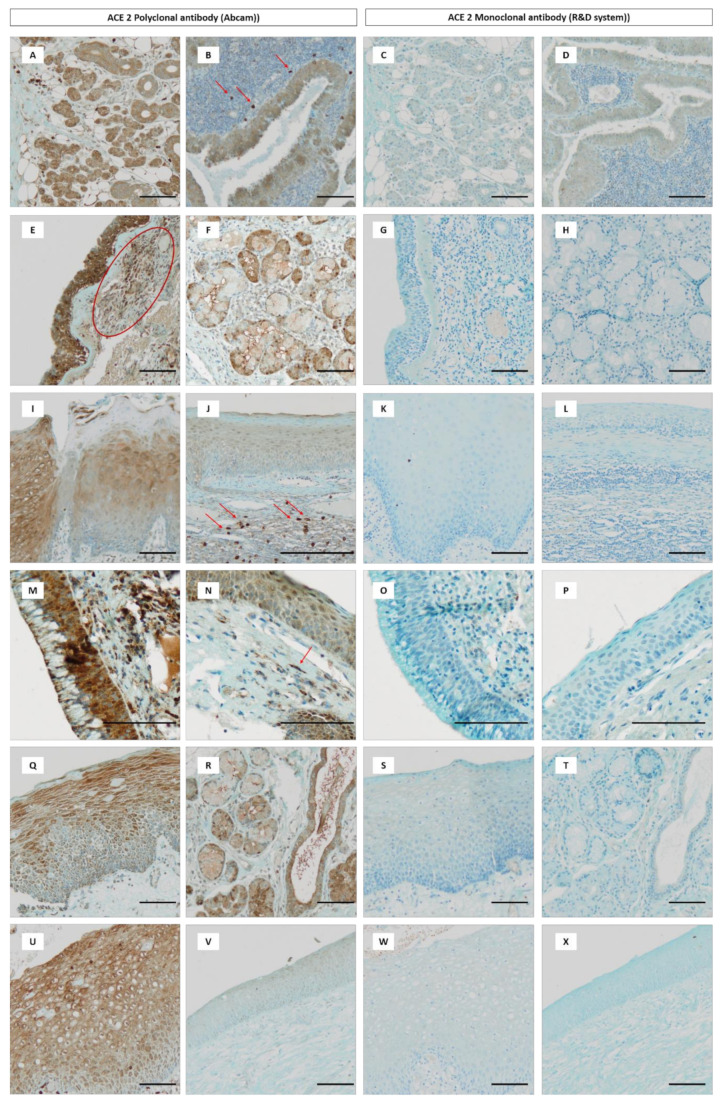
Immunohistochemical labeling of ACE2 in different head and neck tissues. (**A**) Normal salivary gland with ACE2 positive acini and ducts. (**B**) Whartin tumor with positive oncocytic columnar cells; a few inflammatory cells exhibit high expression of ACE2 (arrows). (**E**) Sinus: positive ciliated epithelium, red circle indicated the positivity in inflammatory cells within the stroma. (**F**) Sinus: positive seromucous glands. (**I**) Positive squamous stratified epithelium of lingua (buccal cavity). (**J**) Positive squamous stratified epithelium (weak intensity) of tonsil with some positive inflammatory cells with strong intensity (arrows). (**M**) Positive ciliated epithelium of vocal cord. (**N**) Positive squamous stratified epithelium of vocal cord, arrow indicates positivity in fibroblast. (**Q**) Positive squamous stratified epithelium of larynx. (**R**) Positive seromucous glands of larynx. (**U**) Positive squamous stratified epithelium of oropharynx. (**V)** Faint positivity in squamous stratified epithelium of hypopharynx. (**C**, **D**, **G**, **H**, **K**, **L**, **O**, **P**, **S**, **T**, **W** and **X**) represent the same area than (**A**, **B**, **E**, **F**, **I**, **J**, **M**, **N**, **Q**, **R**, **U** and **V**) but using the monoclonal antibody; there is no positivity in all these locations using this antibody. For all photographs, scale bar = 100 µm.

**Figure 3:**
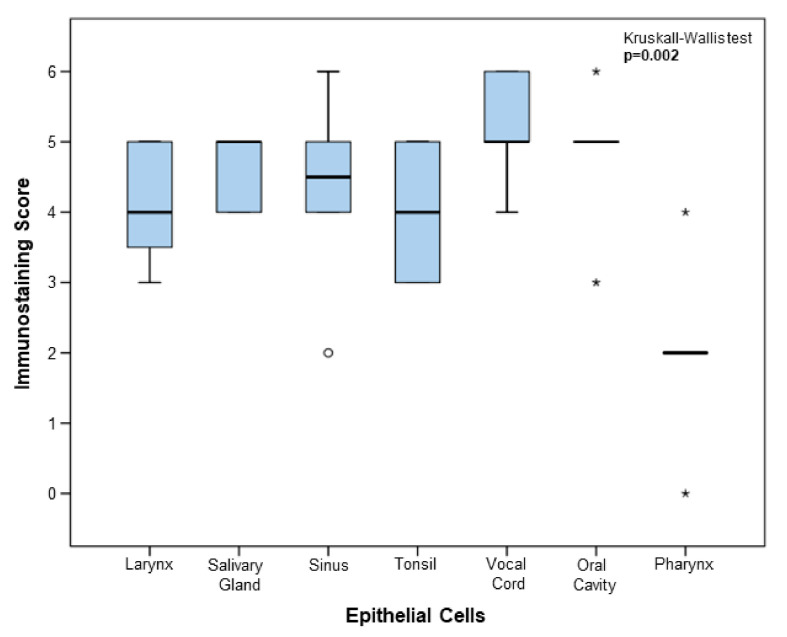
Boxplots comparing immunostaining scores of epithelial cells between each anatomical location. Quantitative analysis of epithelial immunostaining scores in a series of 60 head and neck cases (*p* = 0.002, Kruskal–Wallis test). Open circle shows outliers and asterisks represent extreme values.

Figure 2 depicts the ACE2 labeling with mono- and polyclonal antibodies in each head and neck region and particularly in each epithelial area. Surprisingly, in all instances, no labeling occurred using the monoclonal antibody (Figure 2C,D,G,H,K,L,O,P,S,T,W,X), unlike the polyclonal antibody where a semi-quantitative analysis was achieved (Figure 3).

Among the seven localizations analyzed, the sinus, the oral mucosa, the vocal cord and the salivary gland displayed the highest expression of ACE2 in epithelial cells, with a moderate to strong staining covering more than 75% of the epithelium (Figure 2B,E,I,M). This expression was particularly strong in the ciliated respiratory epithelium of sinuses (Figure 2E) and vocal cords (Figure 2M). However, the staining pattern was weaker and less abundant in the corresponding squamous epithelium of the vocal cords (Figure 2N). Interestingly, a strong and extensive expression profile of glands in the nasal sinus and the larynx is clearly demonstrated (Figure 2F, 2R) and to a lesser extent in pharyngeal glands. In the tonsil and the supraglottic part of the laryngeal squamous epithelia, the mean intensity expression was weak to moderate with a variable staining which can extend from 25 to more than 75% (Figure 2J,Q). Regarding the pharyngeal epithelium, a distinction is observed between the oro- and the hypopharyngeal epithelium, where ACE2 was present in the latter with a weak intensity, covering less than 25% of the epithelium (Figure 2V), whereas oropharyngeal epithelium showed a more extensive and intense expression (Figure 2U). In some cases, an absence of labeling was also observed. Additionally, we observed a wide and weak to moderate staining in ducts and acini from salivary glands (Figure 2A).

The score of immunostained epithelial cells was finally compared between each localization according to the Kruskal–Wallis test, and the score was found to be statistically lower in the pharyngeal region compared to the other ones (Kruskal–Wallis test; *p* = 0.002) (Figure 3). In addition, according to recently published results where a correlation between the age of patients and the symptom of anosmia is reported, we investigated the presence of this correlation in our samples. As shown by Spearman’s Rho test, there was no significant positive correlation between the expression of ACE2 in epithelial cells and the age of patients (Figure S2) (Spearman Rho test; n = 42, median = 53, range from 16 to 82, *p* = 0.65).

### 3.3. ACE2 Expression in Blood Vessels

A weak but abundant staining was seen in blood vessels of oral cavity and supraglottic larynx while this intensity was weaker or absent in vocal cords, salivary glands, tonsils and pharynx, with 25% of labeled cells in mean. The endothelial cells localized in the sinus moderately expressed ACE2 on a surface, covering more than 75% of the endothelium (Figure S3). In some cases, we observed a lesser extent of labeling ranging from 25 to 75%. 

### 3.4. ACE2 Expression in Immune Inflammatory Cells and Fibroblasts

In general, we observed in all anatomical sites a weak but extensive labeling of inflammatory cells, similar to T lymphocytes, macrophages and even neutrophils. This expression was intense in a few cells of tonsils and salivary glands (Figure 2B,J). Interestingly, ACE2 was also weakly detected in fibroblasts in almost all tissues studied. Indeed, we noticed a positive staining of fibroblasts in sinuses, oral cavity and laryngeal mucosae.

### 3.5. ACE2 Expression Correlation with Some Clinical Characteristics

The ACE2 expression score was also evaluated according to the clinical data of several patients (n = 52), such as gender, tobacco consumption, diabetes, arterial hypertension and blood pressure medication (Table 2). Such parameters have been suggested to affect ACE2 expression, but using the non-parametric Mann–Whitney test, we did not find any statistical relation between ACE2 expression in epithelial cells and the clinical parameters (Table 2).

## 4. Discussion

Intensive investigations of ACE2 are currently underway, since it has been confirmed as the host receptor of SARS-CoV-2. Although already studied in many organs, several unanswered questions remain regarding the expression of ACE2 in the head and neck region despite its importance as a suspected entry route for infection. Understanding ACE2 expression in SARS-CoV-2 sites of entry seems imperative in order to select appropriate clinical samples for early virus detection.

**Figure 4:**
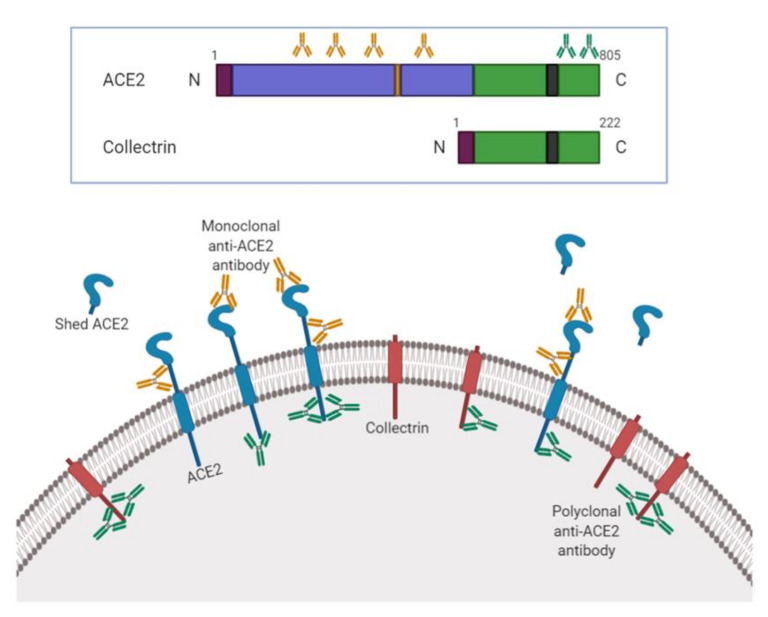
The domain structure of ACE2 and collectrin. Both proteins are type I transmembrane glycoprotein with a signal peptide sequence (purple), a transmembrane domain (black) and a short C-terminal cytoplasmic domain. ACE2 has an extracellular amino-terminal catalytic domain with one zinc binding motif (HEXXH) (orange) corresponding to the active site of the enzyme. The carboxy-terminal cytoplasmic domain of ACE2 share 48% sequence homology with collectrin. The monoclonal anti-ACE2 antibody recognizes a predicted sequence in the N-terminal extracellular domain between Gln18 and Ser740, while the polyclonal antibody targets a precise sequence in the intracellular C-terminal domain corresponding to the 788-805 amino acids located within the homology region denoted by the green box.

In this study, we examined the immunohistochemical expression of ACE2 using two independent antibodies in 70 head and neck tissues (Table 1). To the best of our knowledge, this research is the first to investigate immunohistochemical expression and location of ACE2 across various sites of the upper aerodigestive tract. Remarkably, results obtained were considerably different, so much that no labeling was detected by the monoclonal antibody, unlike the polyclonal antibody. As we validated the specificity of each antibody, several hypotheses can be proposed to explain this discrepancy. As illustrated in Figure 4, the two selected antibodies do not target the same protein sequences. The polyclonal antibody, which revealed a specific signal, targets sequences of ACE2 in its intracellular domain (amino acids 788 to 805 in C-terminal) that share 47.8% sequence identity with C-terminal regions of collectrin, a non-catalytic protein with a small extracellular domain [23]. Cautions must therefore be taken regarding the interpretation of the signal observed because of structural similarities with this novel homolog of the intracellular domain of ACE2 [23,24]. The physiological functions of collectrin have not yet been fully characterized, but interestingly, it has recently been demonstrated that it participates in the formation of primary ciliated cells, which are found in the respiratory epithelium of the nasal cavities, as well as in the transport of amino acids [25,26]. Indeed, just like for ACE2, the main apical membrane transporter, B(0)AT1, requires the presence of collectrin to exercise its functions [27]. Additionally, it has also been shown that these catalytic functions are exerted by collectrin and/or ACE2 [28]. Regarding the monoclonal antibody, it targets a sequence located in the large N-terminal extracellular domain, between amino acids 18 and 740 (Figure 4). Given the growing incidence of studies reporting the presence of ACE2 in the nasal, oral cavity or in the salivary glands, it was essential to understand the absence of labelling with this antibody. Our second hypothesis is based on the existence of a soluble form of ACE2 and therefore the presence of a residual truncated ACE in the apical membrane of such cells. Indeed, several mechanisms could explain the reduction of the expression of the protein at the apical level, such as a reduction in transcription or translation. However, this idea does not correlate with numerous studies demonstrating the expression of ACE2 mRNA in the human airways. During the first epidemic of coronavirus, an in vitro study had shown that the protein ADAM17 was responsible for the cleavage of ACE2 [29]. Later, researchers investigated this cleavage in the airway epithelium and confirmed the involvement of this metalloproteinase in the cleavage of ACE2 [30]. Remarkably, they have demonstrated that this soluble form remains enzymatically active and partially inhibits the entry of SARS-CoV into cells through a “sponge” effect and therefore limits infection. It is easily assumed that the binding of the virus to the soluble form drastically reduces entry, and therefore replication, in cells. In addition, they also located the protein cleavage site, namely between amino acids 716 and 741, corresponding to an area targeted by our monoclonal antibody. The cell-associated form of ACE2 is therefore necessary for infection [30]. The cleavage of the ectodomain of ACE2 may be consider as a potential explanation for the lack of staining but given the complexity of this disease, our next-step investigation will explore this shed ACE2 in sera and nasal secretions from COVID19+ patients. 

COVID-19 presents a broad symptomatology with significant variations in severity, reflecting its complexity. As recently demonstrated, the response of the host’s immune system to SARS-CoV-2 is different depending on gender and age, which could partially explain this distinction in the severity of post-infection disease and in clinical outcome [31]. Four immune signatures were analyzed, corresponding to CD8+ T cells, B cells, NK cells and interferon response. Correlations between ACE2 expression and immune signature enrichment were distinct according to the tissue origin as well as sex of patients highlighting the potential implication of the immune system [31]. Regarding the otolaryngological symptoms in COVID19, a question arises as to the mechanism leading to anosmia. Indeed, several studies, including ours, have recently reported loss of taste and smell to be highly prevalent [5,16,17]. We would therefore expect to observe a major expression of ACE2 in the upper aerodigestive tract. Moreover, a study recently demonstrated a relationship between the age of patient and the expression of ACE2 in the nasal epithelium where RNA expression was significantly lower in children under 10 years old, highlighting why SARS-CoV-2 infection and anosmia symptoms are less prevalent in children [32]. This study may explain why Sars-CoV-2 infection is less prevalent in children. Conversely, Li et al. did not report any differential expression between younger and older persons from 31 normal human tissues analyzed [31]. We therefore evaluated the differential expression of ACE2 in epithelial cells of our different locations according to the age of patients, but we did not find any correlation between ACE2 protein expression and age (Figure S2). However, it will be interesting to evaluate the expression of ACE2 in a cohort of younger patients (<15 years) in order to be able to compare the difference on a more complete panel of ages. Furthermore, it has been shown that many factors and comorbidities could affect ACE2 expression. Indeed, patients with severe acute respiratory distress syndrome present a higher proportion of comorbidities such as arterial hypertension and type 2 diabetes that negatively correlate with clinical outcomes [33]. Many studies have explored the correlations between ACE2 expression and various clinical variables and demonstrated its upregulation in patients presenting pneumonia hypertension and type 2 diabetes, as well as in smoking patients [34,35]. Sex and age are reported to be correlated with ACE2 expression in some tissues [34]. Among our patient population, we did not observe any relation between such clinical variables and ACE2 expression (Table 2).

Several authors have recently focused on the expression of ACE2 in the olfactory epithelium and they identified its expression in sustentacular/non-neuronal cells as well as in neuronal stem cells of the olfactory bulb in a lower proportion [36,37]. Bilinska conducted an animal study by combining different techniques targeting RNA to determine the expression of ACE2 and they also confirmed that the protein was expressed in the sustentacular cells of the olfactory epithelium [38]. In addition to these three teams, Chen and colleagues precisely identified the cellular location of the SARS-CoV-2 receptor in the human airway by analyzing expression at the protein level, so that immunofluorescence revealed an enrichment of ACE2 in the neuroepithelium compared to nasal and tracheal epithelial cells [39]. Additionally, our team has just finalized olfactory cleft radiological evaluations on COVID-19- related anosmia patients. Interestingly, they demonstrated partial or total opacification of the olfactory cleft in some, but not all patients with COVID-19 related anosmia, corresponding to edema and inflammation in the neuroepithelium region [40]. Although the cellular mechanism of olfactory loss remains misunderstood, these observations suggest a potential viral entry through this specific tissue, which should continue to be investigated in the future.

Our immunohistochemical findings reported moderate and expanded staining of ACE2 in epithelial cells, particularly in nasal and oral mucosae, salivary glands and laryngeal glottic area (e.g., vocal cords) (Table 3) and a weaker distribution in tonsil, pharyngeal and laryngeal supra-glottic epithelia. According to published studies, epithelial cells of human upper airway are demonstrated to express high levels of ACE2, especially in nasal epithelial cells including goblet and ciliated cells [41,42]. Moreover, Jia et al. have already demonstrated the predominant apical distribution of ACE2 in differentiated airway epithelia [43]. Apart from these few studies that carried out protein level analyzes, most authors have generally focused on the expression of ACE2 mRNA by various techniques including mainly RNA sequencing or single-cell RNAseq. The presence of ACE2 in respiratory epithelial cells was frequently established through non-staining procedures [44,45]. In the buccal epithelium, positive staining was reported through immunohistochemistry and single-cell RNAseq. As we have observed, Bertram et al. also reported a weakly positive ACE2 expression in tonsillar epithelium but stronger in oral epithelial cells [42] as well as Hamming et al. and Liu et al. who demonstrated a positive labeling in the epithelium lining of human oral epithelium and naso-, oro- and laryngopharynx of seven Chinese rhesus macaques, respectively [14,19]. A previous study also conducted single cell RNAseq analysis based on public data sets and validated the expression of ACE2 in oral epithelial cells, suggesting a new potential route of infection [18]. Finally, interesting results recently confirmed that ACE2 expression was higher in the nose compared to the lower respiratory tract. Nevertheless, it is important to point out that the relative level of ACE2 in the respiratory epithelium of the nasal cavity is described as being low [46], and that several authors agree that ACE2 is a weakly expressed gene [36,47,48]. Therefore, this serious information invites us to remain cautious about the level of translation and expression of the protein. Another point to consider is the effect of tumor microenvironment on ACE2 protein expression in some head and neck locations where the epithelia analyzed may be derived from tumor samples. It has recently been reported that in some cancers from different tissue types, the gene expression of ACE2 was significantly higher in normal tissues compared to matched tumors [49]. Further studies will be required to identify potential factors that may contribute to this change.

Curiously, we found inflammatory cell expression in all head and neck locations, and fibroblasts in almost all sites, but with variable intensities of ACE2 staining, which mainly correspond to lymphoid cells or even macrophages. These findings are sometimes controversial in the literature where some authors revealed different levels of ACE2 expression in T cells and macrophages [18,42,50] while others who did not find any staining in immune cells [14]. However, it was recently discussed that ACE interferes with adaptative immunity by activating macrophages [51], highlighting potential links between this enzyme and immune cells. The same has been reported regarding fibroblasts, with some authors having detected ACE2 expression [14,18], while others report an absence of labeling [52]. Interestingly, the high expression also being observed in salivary glands raises many questions, especially in relation to the possible role of saliva in diagnosis and transmission in Covid19. Some expression of ACE2 in minor salivary glands had been detected previously, notably in SARS-infected epithelial cells [19,20,53]. These data should prompt reflection and bring new interest to the involvement of saliva and salivary glands in studies conducted on COVID19.

Compared to transcriptomic analyzes, immunohistochemistry brings additional important spatial information in tissue samples, but caution must be taken regarding antibody selection. Their specificity is a big challenge to ensuring the reproducibility of antibody-based studies. In 2016, a report from the International Working Group for Antibody Validation (IWGAV) proposed five scientific approaches to validate antibody specificity [54]. In addition, it seems essential to enlarge and diversify patient cohorts and to combine transcriptomic and proteomic strategies, as well as colocalize different markers of SARS-CoV-2 receptors, such as ACE2 and TMPRSS2, to provide an accurate representation of ACE2 expression through all head and neck areas of the whole population.

## 5. Conclusions

We reported for the first time the ACE2 landscape in multiple regions of the upper aerodigestive tract. This research adds a stone to the understanding of the 2019-CoV infection, highlights new potential entry gates, provides new hypotheses regarding the levels of expression of ACE2 and brings new recommendations on the detection of this key protein. Further studies are still required to explore the involvement of ACE2 and determine its precise location throughout the head and neck region.

## Figures and Tables

**Table 1:** Sample characteristics.

Localization	Number of cases (n)	Pathology	Lesion-free Epithelia	Pathologic state
Sinus	10	Chronic Rhinosinusitis	Respiratory	Benign
Tonsil	10	Recurrent tonsillitis or snoring	Stratified Squamous	Benign
Salivary Gland	10	Normal Salivary Gland and/or Whartin tumor	Acini and Ducts or Oncocytic columnar cells	Benign
Glottic Larynx (Vocal Cord)	10	Nodules or Polyps	Stratified Squamous/Respiratory	Benign
Supraglottic Larynx	10	Tumor	Stratified Squamous	Malignant
Oral Cavity	10	Tumor	Stratified Squamous	Malignant
Pharynx	10	Tumor	Stratified Squamous	Malignant

**Table 2:** Correlation between patient clinical data and ACE2 expression score in epithelial cells. Clinical data have been obtained for 52 patients.

	N	Median	*p*-Value
**ACE2 score**	61	5 (0–6)	-
**Gender (F/M)**	15/34		0.26
**Tobacco (No/Yes)**	24/23		0.86
**Diabetes (No/Yes)**	42/5		0.15
**Arterial Hypertenstion (No/Yes)**	36/11		0.17
**Blood pressure Medication (No/Yes)**	45/2		0.15

**Table 3:** Semi-quantitative evaluation of ACE2 expression assessed with the polyclonal antibody in different cell types of seven head and neck sites. The number value corresponds to the median obtained with the Kruskal-Wallis test. The ACE2 expression in inflammatory cells and fibroblasts was reported as positive (+), negative (-), or positive with a strong intensity (++).

	Epithelial Cells	Endothelial Cells (Vessels)	Glands	Inflammatory Cells	Fibroblasts
**Sinuses**	5	4	5	**+**	**+**
**Tonsils**	4	0	0	**++**	**-**
**Salivary Glands**	5	0	0	**++**	**-**
**Oral Cavity**	5	2	0	**+**	**+**
**Pharynx**	2	2	3	**+**	**-**
**Vocal Cords**	5	2	0	**+**	**+**
**Larynx**	4	4	6	**+**	**+**

## Supplementary Materials

The following are available online at https://www.mdpi.com/2079-7737/9/8/235/s1; Figure S1: Anatomical description and location of salivary glands and head and neck structures; Figure S2: Correlation between immunostaining scores of epithelial cells and patient age at the time of collection; Figure S3: Immunohistochemical labeling of ACE2 in endothelial cells of sinuses with the polyclonal antibody.
