# Supplementary material for: ACE2 Protein Landscape in the Head and Neck Region: The Conundrum of SARS-CoV-2 Infection
Source: Biology (Basel). 2020 Aug 18;9(8):235. doi: 10.3390/biology9080235 (PMC7465650; doi:10.3390/biology9080235)
Supplement: Supplementary file 1 [file biology-09-00235-s001.zip › Suppl Fig1.pdf]

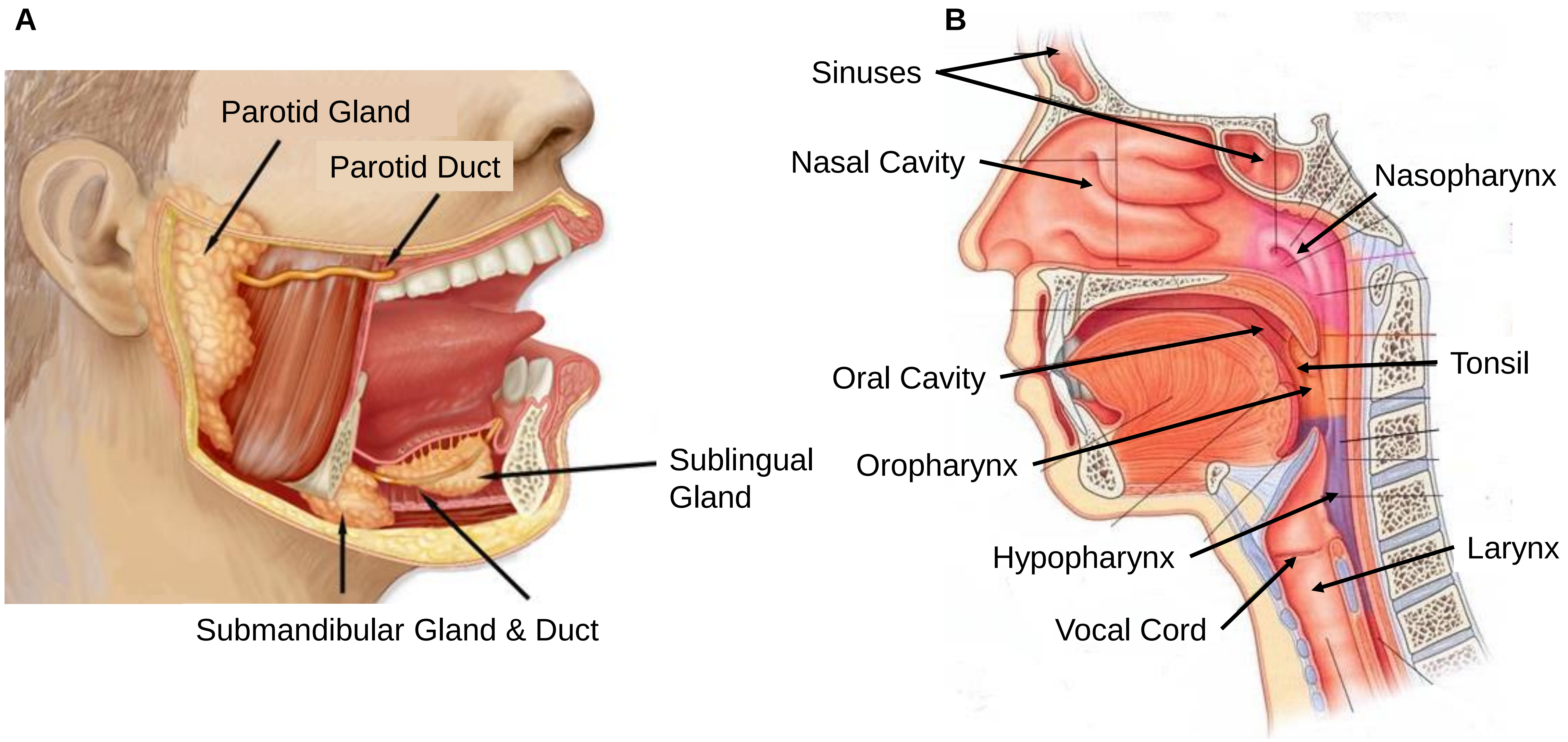

<u>Supplementary Figure 1:</u> Anatomical description and location of salivary glands (**A**) and head and neck structures (**B**).
Schemes were obtained from https://www.parotidsurgerymd.com/ (**A**) and from Gray's Anatomy 2nd Edition, Elsevier 2010 (**B**)
