# Supplementary material for: ACE2 Protein Landscape in the Head and Neck Region: The Conundrum of SARS-CoV-2 Infection
Source: Biology (Basel). 2020 Aug 18;9(8):235. doi: 10.3390/biology9080235 (PMC7465650; doi:10.3390/biology9080235)
Supplement: Supplementary file 1 [file biology-09-00235-s001.zip › Suppl Fig2.pdf]

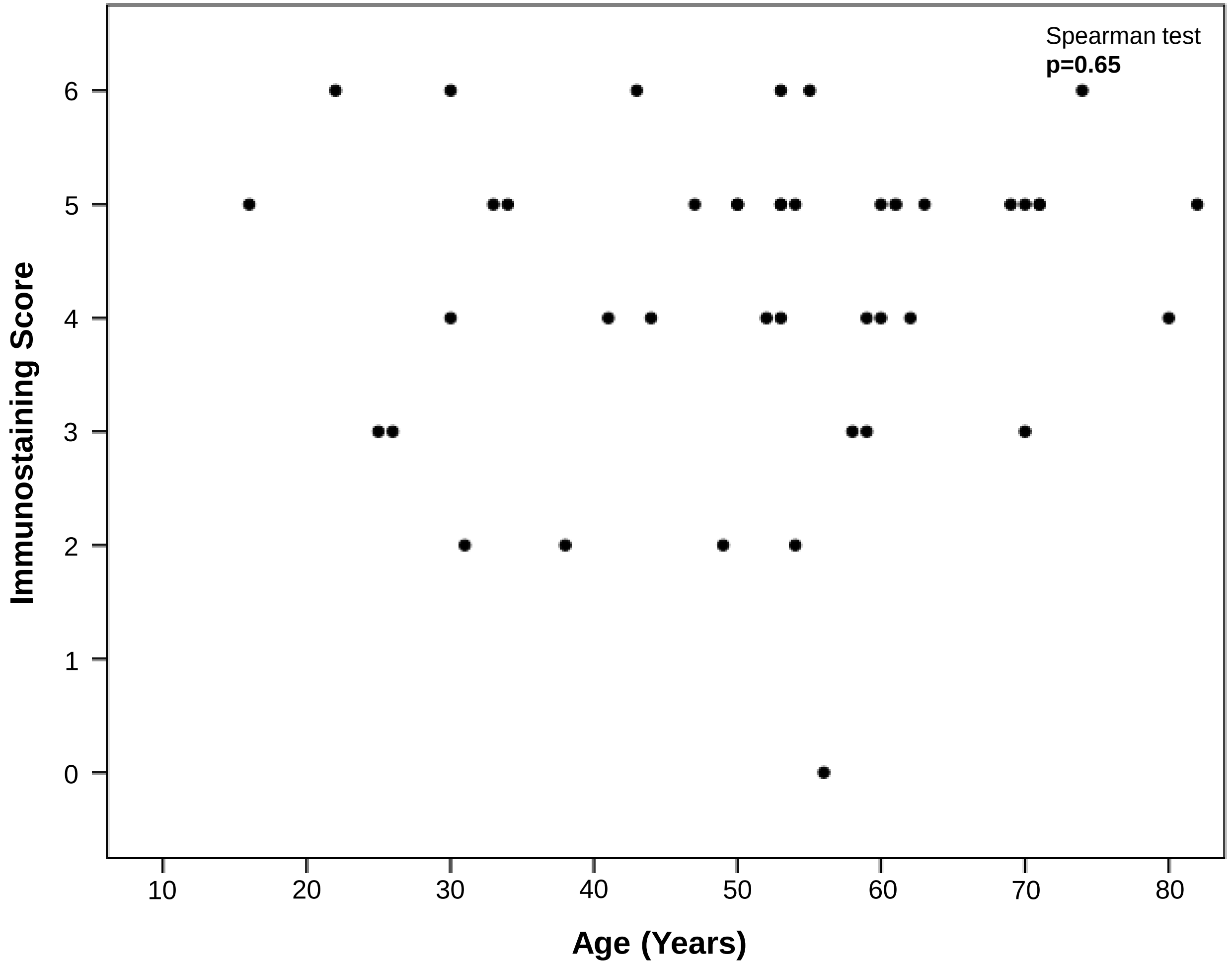

Supplementary Figure 2. Correlation between immunostaining scores of epithelial cells and patient age at the time of collection**.** Spearman correlation test between epithelial immunostaining scores and age of 42 patients (p=0.65, Spearman's rho test).
