# Supplementary material for: ACE2 Protein Landscape in the Head and Neck Region: The Conundrum of SARS-CoV-2 Infection
Source: Biology (Basel). 2020 Aug 18;9(8):235. doi: 10.3390/biology9080235 (PMC7465650; doi:10.3390/biology9080235)
Supplement: Supplementary file 1 [file biology-09-00235-s001.zip › Suppl Fig3.pdf]

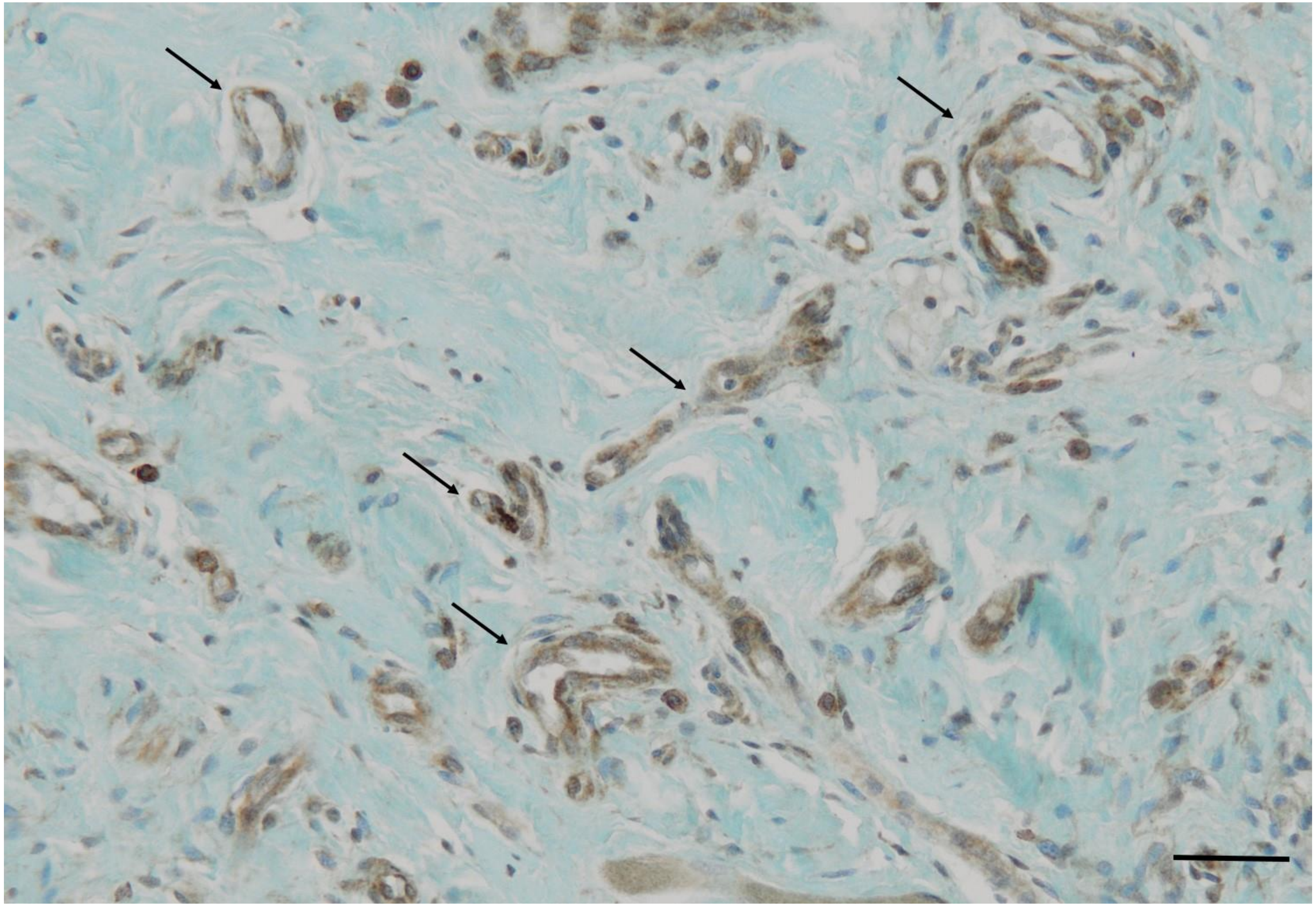

<u>Supplementary Figure 3:</u> Immunohistochemical labeling of ACE2 in endothelial cells (arrows) of sinuses with the polyclonal antibody. Scale bar = 100 µm
